# Gridded daily weather data for North America with comprehensive uncertainty quantification

**DOI:** 10.1038/s41597-021-00973-0

**Published:** 2021-07-23

**Authors:** Peter E. Thornton, Rupesh Shrestha, Michele Thornton, Shih-Chieh Kao, Yaxing Wei, Bruce E. Wilson

**Affiliations:** Environmental Sciences Division, Oak Ridge National Laboratory, Oak Ridge, TN USA

**Keywords:** Environmental sciences, Climate sciences

## Abstract

Access to daily high-resolution gridded surface weather data based on direct observations and over long time periods is essential for many studies and applications including vegetation, wildlife, soil health, hydrological modelling, and as driver data in Earth system models. We present Daymet V4, a 40-year daily meteorological dataset on a 1 km grid for North America, Hawaii, and Puerto Rico, providing temperature, precipitation, shortwave radiation, vapor pressure, snow water equivalent, and day length. The dataset includes an objective quantification of uncertainty based on strict cross-validation analysis for temperature and precipitation results. The dataset represents several improvements from a previous version, and this data descriptor provides complete documentation for updated methods. Improvements include: reductions in the timing bias of input reporting weather station measurements; improvement to the three-dimensional regression model techniques in the core algorithm; and a novel approach to handling high elevation temperature measurement biases. We show cross-validation analyses with the underlying weather station data to demonstrate the technical validity of new dataset generation methods, and to quantify improved accuracy.

## Abstract (summary)

**Table:** 

Measurement(s)	temperature of air • precipitation • shortwave radiation • vapor pressure of air • snowpack
Technology Type(s)	digital curation
Factor Type(s)	elevation • geographic location
Sample Characteristic - Environment	land surface
Sample Characteristic - Location	North America

Machine-accessible metadata file describing the reported data: 10.6084/m9.figshare.14538075

## Background & Summary

Gridded weather products are important historical references to support ecological, agricultural, water resources management, and climate change studies, particularly in regions with sparse weather stations and/or intermittent historical meteorological observations. The gridded products provide a spatially and temporally consistent approach to assimilate available weather station data, taking into account the changes in temperature, precipitation, downwelling radiation, and humidity caused by factors such as elevation, prevailing winds, storm tracks, and proximity to large water bodies. Daymet (https://daymet.ornl.gov) is one such gridded weather product, which provides daily minimum and maximum temperature (Tmin and Tmax), precipitation (Prcp), vapor pressure (VP), shortwave radiation (Srad), snow water equivalent (SWE), and day length on a 1 km × 1 km gridded surface for North America and Hawaii from 1980–2019, and for Puerto Rico for 1950–2019. Maintained by the Oak Ridge National Laboratory (ORNL) Distributed Active Archive Center (DAAC), Daymet is presently updated annually, as the previous year’s weather station data become available and reach a status of archive quality.

Daymet was first developed as a research project to provide daily weather driver data for terrestrial biogeochemical modelling applications. The intermountain West in the Conterminous US (CONUS) was used as the study area to develop and test the gridded data product^1^. A CONUS data product (Daymet V1) was developed from that early model and subsequent algorithm improvements^2,3^. The multi-agency North American Carbon Program (NACP) later supported an update of Daymet V2^4^ which included more years and a larger spatial extent. Due to data and algorithm limitations at the time, Daymet V2 was only available for CONUS, Hawaii, Puerto Rico, Mexico, and southern Canada up to 52 degrees North. With the inclusion of additional weather stations and further algorithm enhancement, the spatial coverage of Daymet V3^5^ was expanded to include all of North America.

The new Daymet V4 dataset^6^ presented here provides effective solutions to known issues while taking advantage of the latest station observation datasets. Biases in station observations are identified and corrected, including inconsistencies among stations in time of observation for both temperature and precipitation, and errors related to temperature sensor bias. Independent validation with radar-based precipitation estimates are used to examine the timing of Daymet V4 precipitation. Cross validation analysis is used to quantify and correct biases related to temperature sensors at high elevation stations. Algorithm improvements address issues of out-of-range regression estimates in both temperature and precipitation, and provide increased accuracy and precision in the gridded data products.

## Methods

### Overview

The workflow which results in final Daymet V4 data records consists of five main steps:

1. collection and filtering of input weather station observations and gridded terrain data,
2. generation of primary output variables,
3. generation of secondary output variables,
4. generation of cross-validation statistics, and
5. data file standardization for archiving and data service.

Each of these workflow steps is described in its own section below. While many steps and sub-steps include automated or code-based workflows, there are also significant human interventions between steps, designed for quality assurance/quality control (QA/QC).

### Daymet input data

#### Daily weather stations – source and general methods

The primary Daymet inputs are daily observations of near-surface maximum and minimum air temperature and daily total precipitation from weather stations. Before Daymet V3, it was necessary to retrieve and combine observations from multiple primary data sources. Since Daymet V4, all weather station inputs can now be acquired from the National Centers for Environment Information Global Historical Climate Network Daily database (GHCNd^7^). This simplified workflow was possible due to the large expansion of GHCNd to cover multiple networks in the US, Canada, and Mexico with consistent QA/QC across all input weather stations^8^. We used GHCNd V3.26, released in April 2019 in Daymet V4.

**Fig. 1:**
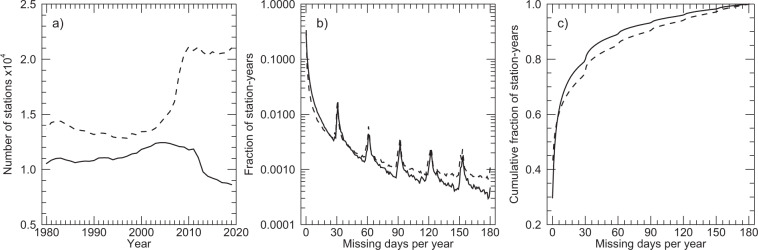
(**a**) Number of stations included in Daymet V4 by year, (**b**) frequency distribution of number of missing days per station-year, and (**c**) cumulative frequency of station-years with increasing numbers of missing days. Frequency distributions are shown as aggregated for all years. In all plots the solid line is for Tmax and the dashed line is for Prcp. The Tmax and Tmin data are nearly indistinguishable in these plots, so for clarity only Tmax is plotted.

A preliminary screening of GHCNd is performed to identify all available stations in the North American domain. The screening was conducted at each station for each of the primary input variables (Tmax, Tmin, and Prcp) in each year. A station-year is removed if more than 180 days of data are missing within a calendar year for a given variable. A station-year removed for one variable might still be included for other variables if the missing days threshold is not exceeded for those variables. Data with reported GHCNd quality flags were also considered as missing. For a few stations with identical locations but different station identifiers, duplicates were removed. The total number of stations remaining after this screening is higher for Prcp than for Tmax and Tmin over the period 1980–2019 (Fig. 1a), with increases in the Prcp station count after year 2000 due to the growth of the Community Collaborative Rain, Hail and Snow (CoCoRaHs) network, and decreasing number of temperature stations in the recent decade due to a sharp drop in stations from networks in Mexico as well as a decline in the number of stations in the US Cooperative Observer Program (COOP) network. In terms of the frequency distribution of number of missing days per station (Fig. 1b), most of the station-years have low numbers of missing days, with secondary peaks at intervals associated with a pattern of whole months being marked as missing within a station-year (Fig. 1b). The fraction of station-year records with no missing data is higher for Prcp (about 40%) than for Tmax or Tmin (about 30%), while 90% of station-years have fewer than 80 or few than 62 missing days for Prcp and Tmax/Tmin, respectively (Fig. 1c).

**Fig. 2:**
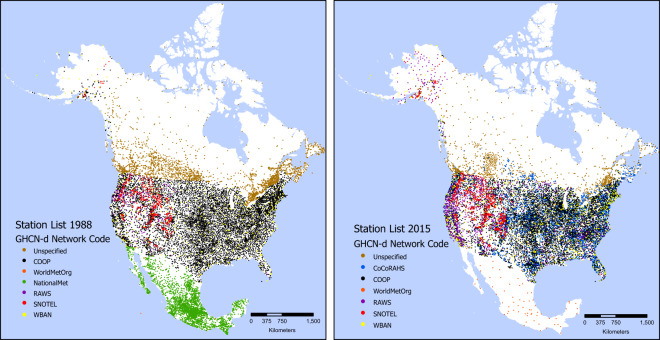
Spatial distribution of annual station data for years 1988 and 2015 based on GHCNd defined station-level Network Code. Further source information is available within the daily data files where embedded flags provide information at the individual station level. The SFLAG1, the source flag, provides up to 30 different values. Most Canadian stations, labeled here as ‘Unspecified’ have an SFLAG1 identifying the source as Environment Canada.

The density of stations varies greatly over the geographic domain, and changes in station networks over time cause shifting patterns of station density (Fig. 2). A primary challenge for the Daymet algorithm is to accommodate spatial and temporal shifts in station density while maintaining as much estimation accuracy as possible across the spatial and temporal domain. Given the geographic separation among North America, Hawaii and Puerto Rico, these three sub-domains were processed independently, using identical methods for each region.

#### Time-of-observation bias corrections

The issue of time-of-observation (TOO) arises from inconsistent report timing among stations. Our objective with Daymet V4 is to standardize the data values for a given calendar day to represent the 24-hour period from midnight to midnight, local time. For some stations, automated sub-daily readings are aggregated to a midnight-to-midnight time frame. Most stations, such as those in the COOP and CoCoRaHS networks, report at some time other than midnight. Combining stations with different reporting times to make estimates at an unmeasured location can lead to estimation biases. These biases are likely to affect the estimation of Tmax since an observation time before noon will usually assign Tmax from the previous 24 hours to the current calendar day. Daily minimum temperature is less likely to be shifted by an observation time before noon since most daily minimum temperature values occur between midnight and sunrise. Daily total precipitation presents a more difficult challenge since any observation period other than midnight-to-midnight will result in misplacement of some amount of precipitation between days. While this timing bias does not affect the overall distribution and long-term climatology and was not explored in earlier versions of Daymet, it may create issues for more time-sensitive applications such as flood and heat wave analysis. Therefore, in Daymet V4 we assessed TOO for each station, as recorded in GHCNd or inferred from other network metadata records. Corrections to the station-level inputs for TOO biases are performed for Tmax and Prcp

##### Maximum daily temperature (Tmax)

For Tmax, we posited that a bias exists in the actual day of maximum temperature for readings that have a TOO before local noon^9^. Previous studies^10,11^ have demonstrated that a bias exists due to the discrepancy in the observation time and actual occurrence of the daily maximum temperature. It is conceivable that for most days, a maximum temperature recorded in the morning is likely the maximum temperature from the previous day. To correct this bias, for GHCNd stations (such as most COOP) that have a TOO prior to noon local standard time (LST), we shifted the daily Tmax values to the previous day. Although TOO is not available for Canadian GHCNd stations, Mekis *et al.*^12^ describe that the majority of manual Canadian climate stations dataset (called the ordinary climate stations) typically have a morning reporting time at 0700 LST and the recorded values have been assigned to the previous calendar day^10^ before the data are incorporated in GHCNd. We therefore did not apply additional TOO bias corrections to the Canadian stations. In addition, all other stations without reported TOO, including stations from Mexican networks, were not adjusted.

To determine if the time adjustment improved Tmax estimates, we compared the cross-validation estimates of the adjusted maximum temperature values to estimates generated from the same V4 algorithm, but with the input observed maximum temperature value day unaltered. We found that shifting the maximum temperature data in this way resulted in substantial reductions in cross-validation error for the daily estimates, and therefore adopted the approach for Daymet V4 processing. See the *Technical Validation* section for detailed results of this analysis.

##### Daily total precipitation (Prcp)

Similar to Tmax, observations of precipitation that are taken once per day but that are not reporting from midnight-to-midnight LST can result in all or part of a precipitation event being attributed to the wrong calendar day. However, unlike Tmax, there is no obvious way to correct the timing issue of Prcp. Our objective was to improve the overall timing of daily precipitation amounts without compromising the frequency of daily precipitation occurrence. We explored two alternative TOO corrections for Prcp, assessing error and bias in estimated precipitation amount and errors in the estimated frequency of wet and dry days for the two approaches. The first method was to move a fraction of the observed daily total precipitation to the previous calendar day, based on TOO at each station. The second method was to shift the entire daily precipitation amount to the previous calendar day based on TOO (same with Tmax). For the first method, the fraction shifted to the previous day was given by the fraction of the 24-hour day elapsed between TOO and the subsequent midnight. The precipitation value of the next calendar day would receive the same treatment in which any fractional amount from this calendar day would be added to the shifted value. For the second method, the total recorded precipitation amount was shifted back one day for weather stations with TOO earlier than noon LST. We obtained TOO information from the metadata of GHCNd, which is available for a majority of stations in the US COOP, Weather-Bureau-Army-Navy (WBAN), Remote Automatic Weather Stations (RAWS), and Snow Telemetry (SNOTEL) networks. Although TOO is not carried into GHCNd distribution for CoCoRaHS stations, the CoCoRaHS training material^13^ indicates that most CoCoRaHS stations have a morning TOO, with 84% of stations recording at 0700 LST. We hence included all CoCoRaHS stations in the TOO adjustment. Given the same reason with Tmax, we did not apply correction to Canadian and Mexican stations, as well as those US stations without TOO information.

We found that the first method (splitting precipitation totals across days) gave the lowest daily errors for precipitation amount but resulted in a large increase in over-prediction of wet-day frequency. The second method (moving entire daily amounts across days) gave an improved daily error for recent years compared to the uncorrected data, and maintained the very low bias in wet-day frequency estimation characteristic of the Daymet methods applied to station data without TOO biases. We therefore selected the second method for Prcp TOO bias correction. Detailed results supporting this choice are provided in the *Technical Validation* section.

#### High-elevation temperature sensor bias correction

Since Daymet V1, gridded estimates of temperature and precipitation in mountainous regions have been aided by the inclusion of the SNOTEL network of high elevation autonomous stations^14^. A data quality issue associated with replacement of temperature sensors beginning in the mid-1990s has been reported^15–17^. The difference between old and new sensors has been shown to be related to the observation temperature, with the new sensors recording higher temperature than the old sensors under colder conditions, and the new sensors recording lower temperature than the old sensors under warmer conditions^16^. Oyler *et al*.^17^ used pair-wise comparisons to perform homogenization of temperature records before and after sensor updates, based on methods by Menne and Williams^18^.

We used the existing Daymet cross-validation framework (described in the *Daymet Algorithms* section, below) to assess patterns of estimation bias in the SNOTEL network before and after the installation of new sensors, using the date of sensor updates for each station from^17^. We found that the pattern of bias was in qualitative agreement with the expected bias patterns previously described. We derived a quantitative relationship between observed temperature and sensor bias using data aggregated across the entire SNOTEL network, and applied that relationship to the observations from older sensors to reduce bias and improve temporal and spatial network homogeneity. The bias correction takes the form of a piece-wise linear function of original temperature measurement (*T*_*original*_), and is applied uniformly to Tmax and Tmin observations for the period prior to sensor update for each SNOTEL station, as follows:

1 $${T}_{{\rm{C}}orre{\rm{C}}ted}=\left\{\begin{array}{l}{T}_{original}+0.4143,\,{T}_{original} < -2{0}^{\circ }{\rm{C}}\\ 1.0245\;{T}_{original}+0.9043,\,-2{0}^{\circ }{\rm{C}}\le {T}_{original} < -5.{3}^{\circ }{\rm{C}}\\ 0.9523\;{T}_{original}+0.5217,\,-5.{3}^{\circ }{\rm{C}}\le {T}_{original} < 3{0}^{\circ }{\rm{C}}\\ {T}_{original}-0.9093,\,{T}_{original}\ge 3{0}^{\circ }{\rm{C}}\end{array}\right.$$

Detailed results leading to this correction are provided in the *Technical Validation* section.

#### Additional gridded surface data inputs and geographic projection

Additional Daymet inputs are a gridded digital elevation model (DEM) and a corresponding land/water mask defining the Daymet domain. The DEM used in Daymet is processed from the National Aeronautics and Space Administration (NASA) Shuttle Radar Topography Mission (SRTM) near-global 30 arc second DEM V2.1. The SRTM DEM was first projected from a geographic coordinate system (GCS_WGS_84) to a Lambert Conformal Conic projection (see^6^ for Daymet projection parameters) and resampled to each 1 km × 1 km Daymet grid. The resampling method used a cubic convolution interpolation with a 1,000 m output cell size. Slope and aspect grids are derived from the DEM within the Daymet algorithm. Horizon files used for radiation estimation were generated separately with the r.horizon model using the GRASS GIS software^19^.

The land/water mask was derived from the MODIS 250 m Land-Water Mask MOD44W_v2^20^. Similar to the DEM, the 250 m MODIS land/water mask was reprojected and resampled to the 1 km × 1 km Daymet grid. Inland water bodies are considered as land in the Daymet domain, retaining only the coastline as the Daymet land/water interface.

### Daymet algorithms

#### A. Spatial and temporal interpolation

The main algorithm to estimate primary Daymet variables (Tmax, Tmin, and Prcp) at each Daymet grid is based on a combination of interpolation and extrapolation, using inputs from multiple weather stations and weights that reflect the spatial and temporal relationships between a Daymet gridcell (measured from its center) and the surrounding weather stations. The approximate number of weather stations used at each Daymet gridcell is defined as a parameter for each Daymet variable. In Daymet V1 this parameter (average number of stations, or *ANS*) was used as an input to an iterative station density estimation algorithm, which produced a search radius that was specific to each gridcell^1^. After a series of algorithm modifications intended to improve robustness in regions of very low weather station density, the Daymet V4 algorithm replaces the iterative station density calculation with a pre-calculated search radius (r_search_) at each gridcell which is sized to capture *ANS* based on arrays of station distance. In regions with relatively high station density, such as most of the CONUS, the search radius approach is nearly identical to the iterative station density approach. In regions with very sparse observation networks, for example in the Arctic regions of Alaska and Canada, the modified approach eliminates artifacts associated with iterations in the previous density calculation, improving the stability of Daymet estimates and reducing mean cross-validation error.

At each Daymet gridcell, a truncated Gaussian convolution kernel is used to assign weights for all surrounding stations identified by r_search_. These weights are held constant until the station list changes, which can happen at the beginning of each year due to weather station data availability. The shape of the Gaussian kernel is defined by a single parameter (the Gaussian shape parameter, or *GSP*). The normalized weight for a given station (with index *i*) in the input weather station list (*w*_*i*_) is given as:

2 $${w}_{i}=\frac{exp\left(-GSP{\left(\frac{{d}_{i}}{{r}_{search}}\right)}^{2}\right)-exp\left(-GSP\right)}{{\sum }_{i=1}^{ANS}{w}_{i}}$$

where *d*_*i*_ is the horizontal distance from a Daymet gridcell to the station. Normalization here ensures that the weights for all stations in the station list for a Daymet gridcell sum to 1.

Because the number and distribution of observations can differ significantly for different meteorological variables, and because the optimal interpolation parameters (ANS and GSP) can differ among variables^1^, we specify a unique pair of ANS and GSP parameters for each of the primary variables (Tmax, Tmin, and Prcp). Station lists and associated weights are calculated at each Daymet gridcell in each year, and for each primary variable. These lists and weights are accessed as inputs to the subsequent workflow steps.

Because the horizontal location information recorded for some stations is not precise enough to assign unique locations within the 1 km grid, we allow for automatic adjustment of station location within +/− 1 km to minimize difference between recorded station elevation and gridded terrain data, as detailed in Thornton *et al*.^1^.

#### Generation of primary output variables

Given the pre-processed weather station inputs and pre-calculated station lists and weights at each Daymet gridcell, two separate workflows are used to produce the primary Daymet output variables: one for Tmax and Tmin and another for Prcp.

##### Daily temperature estimation

Since the workflows for Tmax and Tmin are identical, here we describe them using a generic daily temperature variable T. First, the horizontal coordinates (meters from the origin) at the center of each Daymet gridcell are calculated from the Daymet projected coordinate system^6^. The vertical coordinate, or elevation, (meters above mean sea level) of a target gridcell is obtained from the pre-processed DEM. Horizontal coordinates and elevation of stations are derived from station metadata records for all selected weather stations. Based on the identified weather station list at each Daymet gridcell, the distance (km) and elevation difference (m) between Daymet gridcells and weather stations are calculated.

The estimation of temperature is based on a weighted multivariate regression model which uses the available observations to estimate spatial gradients in the observed temperature in three orthogonal spatial dimensions: two horizontal, and one vertical. This is a modification from the univariate regression adopted in Daymet V1^1^, which included estimates only for the vertical gradient in observations (in this case the temperature lapse rate). By including both horizontal and vertical gradients in a multivariate regression framework, we may obtain additional information about horizontal gradients due to short-term events such as the passage of frontal systems, and due to persistent geographic features, such as nearby water bodies, urban areas, and interaction with large-scale terrain features and prevailing wind directions. Accounting for horizontal gradients removes some aliasing of these effects onto the vertical gradient estimates and improves predictions in regions of both flat and complex terrain.

Because the realization of horizontal and vertical temperature gradients can vary over time, we estimate the gradients separately for each day. To maximize the use of information about spatial gradients provided by the station observations, we use a paired-difference approach as described by^1^ to form the inputs for regressions at each Daymet gridcell. We use a weighted regression model that considers the interpolation weights for each station in the paired difference. Because the paired-difference approach is designed to have a near-zero intercept for the resulting regression equations, we do not include the intercept term in the formulation. Detailed implementation at a single Daymet gridcell is given by the following series of equations.

Paired differences in the station horizontal and vertical positions are given as:

3 $$\left\{\begin{array}{c}d{x}_{p}={s}_{p}\left({x}_{i}-{x}_{j}\right)\\ d{y}_{p}={s}_{p}\left({y}_{i}-{y}_{j}\right)\\ d{z}_{p}={s}_{p}\left({z}_{i}-{z}_{j}\right)\end{array}\right.$$

where *p* is an index from 1 to *np*, with *np* as the number of unique station pairings possible given the station list at each Daymet gridcell, and *np* = (*ANS*^2^*-ANS*)*/2*. The variables *dx, dy, and dz* are the differences in horizontal distances (*x* and *y*) and vertical distance (*z*) between the pair of stations, and the subscripts *i* and *j* denote the index values of each station in the unique pairing from the full station list at each Daymet gridcell. To avoid bias in the mean values of *dx, dy*, and *dz* which can occur if the stations in the list have non-random order with respect to their *x, y*, and *z* coordinates, the variable *s*_*p*_ is introduced, with a value of either 1 or −1 for a given unique pairing *p*, and that value alternating between the two values for each value of *p* from 1 to *np*. As a further guard against biased mean differences, the initial value of *s*_*p*_ for *n* = 1 is switched between 1 and −1. The arrays *dx, dy*, and *dz* form the matrix of independent variables in the multivariate regression model. Since the station list is fixed for all days in a given year, these independent variables are calculated once per year.

At each Daymet gridcell, the regression weight associated with each unique pairing, *rw*_*p*_, is calculated as:

4 $$r{w}_{p}={w}_{i}{w}_{j}$$

where *w*_*i*_ and *w*_*j*_ are the interpolation weights at the estimation point for the two stations making up the unique pairing, from Eq. (2).

Given these regression components, which are the same for each day in a given year at a given Daymet gridcell, temperature difference is calculated for each day as:

5 $$d{T}_{p,d}={s}_{p}\left({T}_{i,d}-{T}_{j,d}\right)$$

where *T*_*i,d*_ and *T*_*j,d*_ are the daily temperature measurements (either Tmax or Tmin) for two stations (*i* and *j*) in a unique pairing, for a given day, *d*.

Based on the inputs defined in Eqs. (2–5), the vector of least squares regression parameters is given in the standard matrix representation for multivariate regression^21^:

6 $$\widehat{\beta }={\left({\boldsymbol{X}}{\prime} {\boldsymbol{WX}}\right)}^{-1}{\boldsymbol{X}}{\prime} {\boldsymbol{Wy}}$$

where the four elements of column vector $\widehat{\beta }$ represent the regression intercept (*β*_0_) and the regression coefficients for two orthogonal horizontal temperature gradients (*β*_1_ and *β*_2_ for gradients in the *x* and *y* directions, respectively) and one vertical (*z* direction) temperature gradient (*β*_3_). ***X*** in Eq. (6) is the (*np* × 4) matrix of independent variables, given as:

7 $${\boldsymbol{X}}=\left[\begin{array}{lccc}1 & d{x}_{1} & d{y}_{1} & d{z}_{1}\\ \vdots  & \vdots  & \vdots  & \vdots \\ 1 & d{x}_{np} & d{y}_{np} & d{z}_{np}\end{array}\right]$$

***y*** is the column vector (*np* × 1) of dependent variables for a given day *d*, given as:

8 $${\boldsymbol{y}}=\left[\begin{array}{c}d{T}_{1,d}\\ \vdots \\ d{T}_{np,d}\end{array}\right]$$

***W*** is the (*np* × *np*) diagonal weighting matrix constructed from the weights in Eq. (4), as:

9 $${\boldsymbol{W}}=\left[\begin{array}{ccc}r{w}_{1} & \cdots \, & 0\\ \vdots  & \ddots  & \vdots \\ 0 & \cdots \, & r{w}_{np}\end{array}\right]$$

***X***′ is the transpose of ***X***, and ()^−1^ indicates the inverse matrix.

Finally, the temperature at the Daymet gridcell location and for the given day *d*, *T*_*est,d*_, is given as:

10 $${T}_{est,d}={\sum }_{i=1}^{ANS}{w}_{i}\left({T}_{i,d}+{\beta }_{1}\left({x}_{est}-{x}_{i}\right)+{\beta }_{2}\left({y}_{est}-{y}_{i}\right)+{\beta }_{3}\left({z}_{est}-{z}_{i}\right)\right)$$

where *x*_*est*_ and *y*_*est*_ are the Daymet gridcell coordinates and *z*_*est*_ is the Daymet gridcell elevation. All geographic units are expressed in meters from map origin or meters above mean sea level, and temperatures are expressed in degrees Celsius.

Two constraints are placed on the estimations generated by Eq. (10). First, *β*_3_ (the vertical temperature gradient) is restricted to the range (−0.012 to 0.001) °C/m. This means that normal temperature lapse rates are limited to at most a 12 °C decrease and 1 °C increase in temperature per 1000 m elevation difference. This constraint reduces spurious estimations in regions of strong topographic relief and very sparse station networks, especially in the far northern extent of the Canadian Rocky Mountains. The second constraint caps any daily temperature estimate from Eq. (10) to no more than 10 °C warmer than the warmest observed temperature in the station list for the given day. This constraint prevents spurious horizontal temperature gradients from causing excessively warm temperatures in regions with very sparse and horizontally skewed station distributions, for example on the southern extremity of the Baja peninsula.

##### Daily precipitation estimation

For Prcp, we also started by calculating the coordinates and distance of all selected weather stations. Since the interpolation parameters ANS and GSP are different for temperature and precipitation, the list of selected stations by Eq. (2) can be different. All daily precipitation observations and gridded estimates are described here in water equivalent units of mm/day.

The precipitation estimation for a given location on a given day is performed in two steps. First, an estimation is made for daily precipitation occurrence (wet vs. dry). Next, for wet days an estimation is made for daily precipitation total. Estimated precipitation occurrence for a given day *d* (*PO*_*est,d*_) is calculated as a binomial variable following Thornton *et al*.:^1^

11 $$PO{P}_{est,d}={\sum }_{i=1}^{ANS}{w}_{i}P{O}_{i,d}$$

12 $$P{O}_{i,d}=\left\{\begin{array}{c}0;\,{P}_{i,d}=0\\ 1;\,{P}_{i,d} > 0\end{array}\right.$$

13 $$P{O}_{est,d}=\left\{\begin{array}{c}0;\,PO{P}_{est,d} < PO{P}_{crit}\\ 1;\,PO{P}_{est,d}\ge PO{P}_{crit}\end{array}\right.$$

where *POP*_*est,d*_ is the estimated probability (range 0 to 1) of precipitation occurrence at a given estimation location on a given day, *w*_*i*_ is the station interpolation weight given by Eq. (2), *PO*_*i,d*_ is the observed precipitation occurrence for a given station *i* in the station list on day *d*, *P*_*i,d*_ is the observed precipitation amount for that same station and day, and *POP*_*crit*_ is a threshold parameter for occurrence estimation.

Contingent on *PO*_*est,d*_ = 1, the weighted multivariate regression framework of Eq. (6) is used to estimate horizontal and vertical gradients in precipitation. For the purpose of estimating spatial precipitation gradients, a temporal smoothing filter is applied to the daily precipitation observations at each station. The filter has a width of 5 days and is centered on the day of estimation with relative filter weights from days *d*-2 to *d* + 2 as [1, 2, 3, 2, 1]. Only wet days (and weights associated with those days) within the filter time window are included in the smoothed value at day *d*. For example, the smoothed value for a filter window with daily precipitation values given by [0, 0.1, 0.2, 2.3, 0.5] would be (2*0.1 + 3*0.2 + 2*2.3 + 1*0.5)/(2 + 3 + 2 + 1) = 0.7375. The filter window width is truncated when the day of estimation is within the first or last two days of the year. Note that the smoothed precipitation values are used to estimate gradients, but final daily precipitation predictions are based on the un-smoothed station observations.

Compared to the regression matrices for temperature gradients, there are two differences for precipitation gradients. First, the regression weight for a unique pairing of stations is set to zero if either or both of the smoothed precipitation inputs for that day are zero:

14 $$r{w}_{p}=\left\{\begin{array}{c}{w}_{i}{w}_{j};P{S}_{i,d}and\,P{S}_{j,d} > 0\\ 0;otherwise\end{array}\right.$$

where *PS*_*i,d*_ and *PS*_*j,d*_ are the smoothed precipitation values at stations *i* and *j* on day *d*. Second, the column vector ***y*** is made up of differences in these unique pairs of filtered precipitation observations as follows:

15 $${\boldsymbol{y}}=\left[\begin{array}{c}dP{S}_{1,d}\\ \vdots \\ dP{S}_{np,d}\end{array}\right]$$

16 $$dP{S}_{i,d}=\left\{\begin{array}{c}{s}_{p}\left(P{S}_{i,d}-P{S}_{j,d}\right);P{S}_{i,d}and\,P{S}_{j,d} > 0\\ 0;otherwise\end{array}\right.$$

where *s*_*p*_ is the sign switching mechanism as described for Eq. (3).

Still contingent on *PO*_*est,d*_ = 1, the daily total precipitation *P*_*est,d*_ at a Daymet gridcell and day *d* is estimated using the weighted sum of wet stations as:

17 $${P}_{est,d}=\mathop{\sum }\limits_{i=1}^{ANS}\left\{\begin{array}{c}\frac{{w}_{i}\left({P}_{i,d}+{\beta }_{1}\left({x}_{est}-{x}_{i}\right)+{\beta }_{2}\left({y}_{est}-{y}_{i}\right)+{\beta }_{3}\left({z}_{est}-{z}_{i}\right)\right)}{{w}_{sum,d}};{P}_{i,d > 0}\\ 0;{P}_{i,d}=0\end{array}\right.$$

where *w*_*sum,d*_ is the sum of all *w*_*i*_ for wet stations on day *d*.

The daily precipitation estimates from Eq. (17) are subject to several constraints. First, the gradients estimated from weighted regression can sometimes result in *P*_*est,d*_ < 0, and those cases are truncated to 0. Second, if *P*_*est,d*_ is more than twice the highest measured precipitation value from the station list for day *d*, those values are truncated at two times of the max measured precipitation value. Third, constraints are placed on the spatial gradients as estimated from Eq. (17), with *β*_1_ and *β*_2_ (the horizontal components of spatial precipitation gradient) constrained to the range (−0.001, 0.001) mm/day/m and *β*_3_ constrained to the range (0, 0.02) mm/day/m. These constraints were determined empirically by examining histograms of calculated gradients and their joint distributions with anomalous precipitation outputs from Eq. (17). Fourth and finally, if precipitation occurrence (via Eq. 12) is recorded for three or fewer stations in the list for a given gridcell on a given day (suggesting a more localized storm), then all values of *β*_1_, *β*_2_, and *β*_3_ are forced to zero. This final constraint prevents some spurious extrapolations near the edges of localized daily precipitation events.

Daymet V3 included an additional constraint limiting precipitation to a maximum of 200 mm/day. This constraint was related in part to limitation imposed by the data storage approach used for earlier Daymet versions to reduce data volumes, and in part to uncertainty on what the legitimate upper boundaries for daily total precipitation should be. We found that, with the other constraints described above, we could remove this artificial upper limit and improve representation of extreme precipitation events without causing spurious high estimations. An example of the impact of this change is provided in the Technical Validation section.

#### Generation of secondary output variables

In addition to Tmax, Tmin, and Prcp, Daymet also includes estimates of other important meteorological variables that are not routinely observed, or are only available at a small fraction of weather stations. These secondary variables include daylight average shortwave radiation (Srad), daily average water vapor pressure (VP), duration of the daylight period (daylength), and a simple estimate of accumulated snowpack, measured as snowpack water equivalent (SWE). The daylength estimate is based on geographic location and time of year. Estimates of other secondary variables (Srad, VP, and SWE) are derived from the primary variables (Tmax, Tmin, and Prcp) based on atmospheric theory and empirical relationships, as described below.

The detailed equations for joint estimation of Srad and VP based on Tmax, Tmin, and Prcp inputs are described in Thornton and Running^2,22^ with modifications as given in Thornton *et al*.^3^. Here we provide a summary of the theory and workflow. Based on the observed positive relationship between diurnal temperature range and daily total atmospheric transmittance originally described by Bristow and Campbell^23^ and further developed by Running *et al*.^24^ and Hungerford *et al*.^22^, daily Daymet Tmax and Tmin are used to estimate daily total transmittance of shortwave radiation at each Daymet gridcell and each day. The parameterization of this empirical relationship is sensitive to regional and seasonal variation in mean diurnal temperature range, to variation in atmospheric attenuation due to decreased overlying air mass with increasing terrain height, diurnal variation in optical thickness due to solar angle, reduction in transmittance with increased humidity, and observed variation in empirical relationships on days with and without precipitation.

In addition to these factors influencing atmospheric transmittance, the fraction of clear-sky transmittance realized on any day is also used to estimate the fraction of incoming radiation received as direct vs. diffuse radiation. The direct beam component is used in conjunction with terrain slope, aspect, and local horizon angles to estimate beam-slope geometry for land surface in flat or complex terrain, including the variation of this geometry over the course of each day on ten-minute time steps. Digital terrain data are also used to estimate the fraction of unobstructed sky visible at each estimation location, to estimate attenuation of incoming diffuse radiation. The influence of snow cover is also considered, as snow-covered land surface interacts with cloud cover to enhance incident radiation through multiple reflection and absorption pathways.

Estimation of water vapor pressure (VP) is based on the observed correspondence between night-time minimum temperature and dewpoint temperature for many climate regimes^22,24^ with improvements to that relationship for arid and semi-arid climates as described by Kimball *et al*.^25^. Because aridity correction requires an estimate of potential evapotranspiration (PET) and PET requires an estimate of incoming shortwave radiation, we use an iterative approach to jointly estimate radiation and humidity, as described in Thornton *et al*.^3^.

Estimated snow water equivalent (SWE) is based on a very simple temperature driven model of snow accumulation and snowmelt, as described in Thornton *et al*.^3^. The sole purpose of the SWE calculation is to provide an approximate control on Srad through the multiple reflection mechanism. We make SWE data available as part of Daymet V4 so that users can accurately diagnose the influence of this snow correction on Srad estimates. We encourage researchers who require a more accurate estimate of snowpack dynamics to use the temperature, precipitation, and potentially radiation and humidity variables from Daymet v4 to drive a more capable and sophisticated snow process model.

#### Generation of cross-validation error estimates

Since the first public release of Daymet, results from a comprehensive cross-validation analysis have accompanied each release of the gridded daily surface weather products. The purpose of the cross-validation analysis is to provide users with details needed to evaluate the fitness of Daymet for each unique application.

The cross-validation analysis treats each variable-station-year of data from the input station lists as a unique record. This means that each primary variable (Tmax, Tmin, and Prcp) is handled separately at each station location and each year. For each such record, estimates of the primary variable are made by dropping that record from the input station list and using the exact estimation methods described above to make estimates for the primary variable on each day of the year. Since each station in the input list can have missing days where the primary variable is not recorded, only days with non-missing data for the cross-validation station record are used to calculate error statistics for that record. The number of missing days in each record is used to provide appropriate weights when reporting multi-station averages or time series summaries for the cross-validation results.

A complete record of the primary variable estimates for each day with non-missing data, for each station and each year, together with the corresponding daily observed values, are made available for user download (citation below). These cross-validation records include all of the relevant meta-data associated with each station, to allow users to assess patterns of error in relation to station location, observation type, station network, year of observation, seasonal patterns, or other analyses as deemed appropriate by the users.

Many different summary statistics can be computed from these paired daily observations and estimates, such as mean absolute error, root mean squared error, or bias. Different time periods can also be evaluated, according to the user needs. In the Technical Validation section below, we focus on mean absolute error and bias for daily and annual time periods. Further details of the Daymet V4 station-level cross-validation results is provided by Thornton *et al*.^26^.

## Data Records

The Daymet V4 data^6^ are available from the NASA-sponsored ORNL DAAC. The data are available in CF compliant netCDF file format for the time period 1980–2019 for the separate spatial extents of Continental North America and Hawaii, and from 1950–2019 for the Puerto Rico/Virgin Islands spatial extent. Data are geolocated in a projected coordinate system, the Lambert Conformal Conic projection, with a spatial resolution of 1 km × 1 km. Data are updated on an annual schedule.

Daymet cross-validation data are also made available through the ORNL DAAC^26^. Each data file contains the daily observations extracted from GHCNd and associated Daymet model predicted primary variables (Tmax, Tmin, and Prcp), based on the cross-validation methods described above, for all input stations across the entire period. Also included are the corresponding station metadata files for each variable and year, including station name, station identifier, latitude, longitude, and elevation.

The Daymet V4 data are available by direct data download as well as specialized tools and services that focus on open, interoperable, and programable access of the Daymet data are shared from the web site https://daymet.ornl.gov/.

## Technical Validation

We provide technical validation details supporting all bias corrections applied to input data from the weather station networks, followed by results from the Daymet V4 cross-validation analysis. We also provide some examples illustrating the Daymet V4 data products.

### Time-of-observation bias corrections

#### Assessing maximum temperature time-of-observation bias correction

To evaluate the influence of a TOO bias on estimates of Tmax, we examined data records from six years: three in the earlier part of the Daymet period (1988, 1989, and 1990) and three from the later part (2015, 2016, and 2017). As described above, we focused on the US COOP network for this analysis, since we have TOO metadata records available for most stations in that network. Among all COOP stations, about 50% of Tmax records in early years and about 75% of Tmax records in later years have TOO before noon LST (Table 1). Given that a significant number of stations are exposed to this source of bias, we expected that the bias correction would have an important and positive impact on the quality of daily Tmax estimates throughout the Daymet period of record.

**Table 1:** Summary of TOO available from GHCNd during the six years of analysis.

year	maximum temperature*			precipitation		
	station days	%TOO before noon	% nodata	station days	%TOO before noon	% nodata
1988	1,729,277	49.53	2.26	2,914,822	56.70	11.90
1989	1,742,252	50.46	2.61	2,942,135	56.94	12.42
1990	1,746,314	51.24	2.91	2,937,209	57.26	12.89
2015	1,693,990	74.90	2.03	6,877,546	83.57	5.50
2016	1,676,722	74.90	2.23	6,860,087	84.68	7.87
2017	1,63,5940	74.90	2.40	6,892,927	84.38	7.40

Only US stations are shown here to illustrate percentages due to widely missing Canadian and Mexican TOO information. The percent of station-days with missing data is given for each variable and each year (% nodata). *US COOP Network Only.

We used the Daymet V4 temperature prediction and cross-validation algorithms as described above to compare cross-validation errors for the six analysis years with and without the TOO bias correction. We found that with the TOO bias correction, mean absolute error (MAE) for daily Tmax estimates dropped significantly in all six years. For the early years the average daily error was 1.81 °C without correction and 1.65 °C with correction and for the later years the same error was 1.62 °C without correction and 1.23 °C with correction, for about a 9% reduction in error for the early years and about a 24% reduction for the later years. Because this TOO bias correction moves observations in the input station dataset by at most one day, we did not expect a significant impact of this TOO bias correction on cross-validation errors based on longer averaging periods such as annual mean Tmax derived from daily estimates. This expectation was correct: MAE for annual mean Tmax was the same with and without the TOO bias correction, at 0.79 °C for the earlier years and 0.55 °C for the later years. The bias in Tmax estimates as determined by daily cross-validation analysis is very small, and is not affected by TOO correction, with Daymet underestimating Tmax on a daily basis by 0.020 °C for the earlier years, and by 0.005 °C for the later years. Cross-validation results for individual years for this evaluation are shown in Table 2.

**Table 2:** The percent change in weighted average daily MAE (dayMAE) and period of record MAE (porMAE) for maximum daily temperature during the selected six years evaluated with the Daymet V4 algorithm through two runs; time-adjusted vs not adjusted input values.

year	Maximum daily temperature (Tmax)			
	%change mean dayMAE	%change mean porMAE	Mean Total nstns	Avg good days/stn
1988	−8.9482	−0.5013	10,666	344.13
1989	−8.8567	−0.3006	10,644	345.03
1990	−9.4025	−0.1938	10,721	345.61
2015	−24.0978	−0.2892	9,124	350.70
2016	−23.6063	−0.2885	8,968	349.75
2017	−24.6029	0.5235	8,712	350.08

#### Assessing precipitation time-of-observation bias correction

We performed a similar cross-validation analysis to evaluate the influence of TOO bias on estimates of Prcp, examining the same six years and comparing error statistics with and without correction. Among all US GHCNd stations, about 56% of Prcp records in early years and about 84% of Prcp records in later years have TOO before noon LST (Table 1). Given that a significant number of stations are exposed to this source of bias, we expected that the bias correction would have an important and positive impact on the quality of daily Prcp estimates throughout the Daymet period of record.

As described in the Methods section, we tested two correction approaches; one shifted fractions of daily precipitation totals and another moved the entire daily total to the previous day. While the fractional shift method produced the lowest daily MAE in cross-validation analysis, it also caused a significant increase in precipitation bias and a small increase in annual total precipitation MAE (Table 3). Daily MAE for the fractional shift approach averaged 1.6 mm/day for earlier years, and 1.2 mm/day for later years, compared to 1.7 mm/day and 1.5 mm/day for the uncorrected data in those two periods, respectively. This represents about an 8% and 18% reduction in daily MAE for the earlier and later years, respectively, using the fractional shift approach. Bias for the fractional shift approach averaged +0.08 mm/day and +0.10 mm/day for the earlier and later years, respectively, compared to +0.04 mm/day and +0.08 mm/day for those same periods using the uncorrected data. This represents an increase in bias of about 76% and 24% for the earlier and later periods, respectively. The whole-day shift correction method daily MAE from cross-validation was 0.18 mm/day and 0.13 mm/day for earlier years and later years, respectively. This represents an *increase* in daily MAE of about 3% for the earlier years, and a decrease of about 9% for later years, compared to the use of uncorrected data. The bias statistics for the whole-day shift approach were + 0.05 mm/day and + 0.09 mm/day for the earlier and later years, respectively. This represents an increase in bias of about 5% and 6% for the two periods, respectively. As expected, and similar to the results for maximum temperature, cross-validation MAE for estimates of annual total precipitation derived from daily estimates was not significantly influenced by TOO bias correction, assessed at about 0.3 mm/day for all cases (uncorrected and both bias correction methods).

**Table 3:** The percent change in weighted average daily MAE (dayMAE), period of record MAE (porMAE), and bias for precipitation during the selected six years evaluated with the Daymet V4 algorithm through two runs; time-adjusted vs not adjusted input values.

year	Precipitation (Prcp): Total daily value shift				
	%change mean dayMAE	%change mean porMAE	%change bias	Mean Total nstns	Avg good days/stn
1988	2.8483	0.6154	0.0000	13,235	349.22
1989	3.2680	0.9585	4.1667	13,048	351.25
1990	3.9652	0.8721	4.6512	12,918	351.77
2015	−8.3990	0.0000	4.5455	20,476	329.20
2016	−7.9365	0.3861	8.0000	20,263	330.39
2017	−9.2350	−0.3774	5.1282	20,340	329.83
**year**	**Precipitation (Prcp): Fractional daily value shift**				
	**%change mean dayMAE**	**%change mean porMAE**	**%change bias**	**Mean Total nstns**	**Avg good days/stn**
1988	−7.6780	2.4615	87.1795	13,235	349.24
1989	−8.4967	3.1949	64.5833	13,045	351.29
1990	−8.0391	2.9070	81.3953	12,918	351.79
2015	−19.0945	2.6515	14.7727	20,467	329.22
2016	−17.8744	3.8610	32.0000	20,265	330.37
2017	−19.2281	2.6415	25.6410	20,338	329.86

The upper section of the table shows results for shifting entire daily precipitation totals, while the lower section shows results for shifting fractions of daily precipitation.

This analysis suggests that the two bias correction methods represent a compromise, with the fractional shift approach representing a more realistic transfer of precipitation between days, but requiring some additional information to prevent an increase in number of wet days and precipitation bias when a single wet day is split across the daily measurement boundary. By not splitting daily events, the frequency of wet and dry days is retained from the original data (results not shown) and the growth in bias is mitigated, but the improvements in daily estimates are smaller and inconsistent in time. We decided to use the whole day shifting approach to avoid shifting the precipitation frequency distributions and increasing bias.

To provide further confidence that the time shifting approach for precipitation was warranted, we performed a correlation analysis of the timing of Daymet results with and without time-shifting correction using the radar-based Stage IV Quantitative Precipitation Estimate (ST4^27^). The ST4 data merges raw radar-based precipitation estimates with automatic hourly rainfall gauge observations and is further quality controlled by several National Oceanic and Atmospheric Administration (NOAA) River Forecasting Centers (RFCs). ST4 is available at hourly time step in 4-km horizontal resolution since 2002. An evaluation performed by Gourley *et al*.^28^ suggests that ST4 has the highest correlation coefficient with gauge observations among various gridded precipitation products. By aggregating 24-hour ST4 at different starting time, we try to identify when the highest correlation coefficient between Daymet and ST4 can be reached and use it to indirectly assess the actual timing of daily precipitation estimates from the Daymet algorithm. This analysis goes beyond the cross-validation approach, by using the full gridded Daymet outputs as opposed to only the Daymet predictions at individual surface observing stations. We used the Daymet V3 outputs^5^ to assess timing of precipitation without TOO bias correction, and the Daymet V4 outputs (using whole-day shifting approach) to assess timing of precipitation with TOO bias correction.

At each Daymet grid point, the nearest ST4 grid point was first identified. The 24-hour ST4 was then aggregated from t = −23, −22, …, 23, in which t represents the starting hour before (positive) or after (negative) midnight LST. For instance, t = 17 indicates that the 24-hour ST4 is calculated from 7AM previous day to 7AM current day, which is the reporting time for most GHCNd stations. To avoid further complicating the problem, daylight saving adjustment is not considered.

**Fig. 3:**
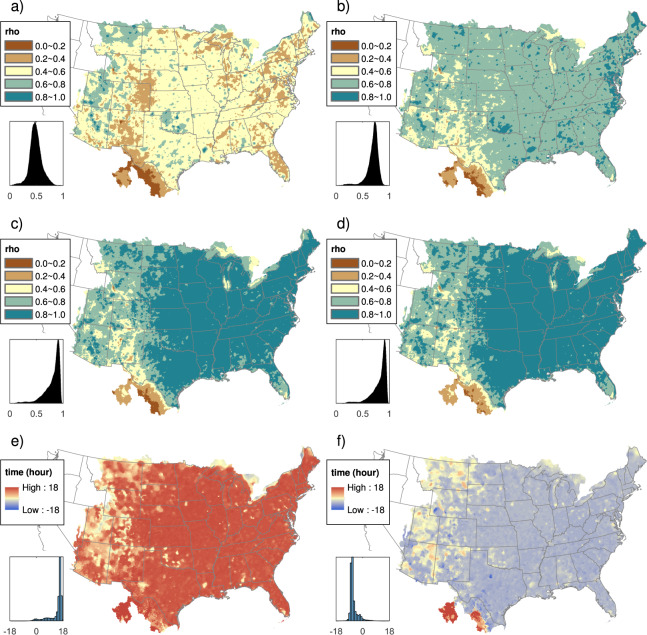
Analysis of Daymet precipitation timing by 2010–2019 hourly ST4 data. Panel (**a**) shows the correlation coefficient (rho) between Daymet V3 and 24-hour ST4 aggregated from local midnight to midnight. By testing different ST4 aggregation timing, panel (**c**) shows the maximum correlation coefficient between Daymet V3 and 24-hour ST4, and panel (**e**) shows the corresponding timing. Panels (**b**), (**d**), and (**f**) are similar to (**a**), (**c**), and (**e**) but calculated by Daymet V4.

Figure 3(a) shows the correlation coefficient between Daymet V3 and 24-hour ST4 aggregated from midnight to midnight LST. Clearly, since most of the daily stations do not report from midnight to midnight, the correlation coefficient is weak and less than 0.5 for most grid points. By testing different ST4 aggregation timing, Fig. 3(c) shows the maximum correlation coefficient between Daymet V3 and 24-hour ST4, and Fig. 3(e) shows the corresponding timing. In terms of the maximum correlation, it is greater than 0.9 in the majority of the eastern US. The correlation declines in the western US where we have serious radar blockage issues. It also shows low correlation in Mexico and above the Great Lakes where we may not have high quality gauges records for both Daymet and ST4. Figure 3(e) suggests that the timing of Daymet V3 daily precipitation is mostly earlier than midnight and peak at t = 17, which is consistent with the timing of most GHCNd stations.

By repeating the analysis using Daymet V4, the corresponding results are showed in Fig. 3(b,d,f). In terms of maximum correlation, the results of Fig. 3(d) are similar to Fig. 3(c), with some slight improvement. Except for some parts of the western US and Mexico, the timing of Daymet V4 daily precipitation is later than midnight and peak at t = 7. Since the daily timing is now pushed closer to midnight, stronger correlation is shown in Fig. 3(b), as expected. This analysis corroborates that the whole-day shifting approach has improved the timing bias, moving the Daymet results closer to a local midnight-to-midnight measurement, albeit shifting from a large early bias to a smaller late bias. The fact that this compromise is an overall improvement in timing is supported by the generally higher correlations shown for V4 than for V3, comparing Fig. 3(b) with Fig. 3(a).

### Assessment of SNOTEL temperature sensor correction

We used the Daymet cross-validation framework to quantify the relationship between observed temperatures before and after sensor replacement in the SNOTEL network of high-elevation stations, and to estimate any empirical relationship between those temperature differences and observed temperature. We examined the difference in cross-validation prediction bias at each SNOTEL station before and after sensor replacement, as a function of observed temperature. The theory of this analysis depends on the existence of a large number of stations outside the SNOTEL network (mainly the US COOP stations) but within the same geographic region. We assume that the instrumentation within the non-SNOTEL networks is consistent over time, and that the cross-validation estimates at each SNOTEL station location will be significantly influenced by the presence of the non-SNOTEL network stations. Comparing estimates so derived with the SNOTEL station observations (as per the cross-validation protocol) will result in a characteristic pattern of bias at the SNOTEL measurement site. If the change in SNOTEL instrumentation causes a systematic shift in the observed temperature at the station, then this pattern of cross-validation bias should shift accordingly when examined before and after the sensor change. Furthermore, since one sensor is used for measurement of minimum and maximum temperature at the SNOTEL station, we expect that the differences in cross-validation bias patterns, such as exist, should overlap for the part of the temperature range with significant overlap in the measurement temperatures.

**Fig. 4:**
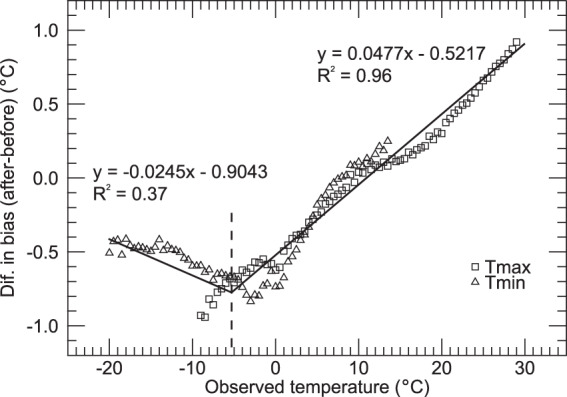
Piece-wise linear regressions based on combined Tmax and Tmin bias differences. Each symbol represents the difference between daily cross-validation bias values after vs. before sensor replacement, for a single variable (Tmax or Tmin) and for a binned sample of observed temperatures taken across all SNOTEL stations. Observed temperature bins are 0.5 °C wide. Solid lines show the piece-wise regression fits, and dashed line shows the piece-wise break-point at −5.3 °C.

We aggregated cross-validation results from all SNOTEL stations and binned them by measurement temperature and found that there are distinguishable patterns of cross-validation bias for both Tmax and Tmin observations. Examining the differences in bias before and after sensor replacement at each station, we found a coherent and nearly linear pattern of bias differences in both Tmax and Tmin cross-validation results over the range of observed temperature from about −5 °C to 30 °C (Fig. 4). The same linear pattern extended down to observed temperatures of −15 °C for Tmax, but not for Tmin (Fig. 4). Based on these results, we estimated bias corrections using one piece-wise regression fit for observed values from 30 °C down to a break-point at −5.3 °C, and a second fit for values from −5.3 °C to −20 °C (Fig. 4). For values above 30 °C and below −20 °C, we held the bias correction constant as given by the relevant piece-wise fit at those values.

We used a synthetic analysis of station data from a cold region outside the SNOTEL domain to assess whether our approach can provide a bias correction for a known (synthetic) imposed bias in a subset of the station data. We found that the cross-validation bias method gave a reliable estimate of the known synthetic bias pattern (results not shown).

### Summary of Daymet V4 cross-validation results

Complete cross-validation results for Tmax, Tmin, and Prcp are provided with the Daymet V4 data release, for every input weather station and every estimation day over the 40-year period. Users are encouraged to consult the cross-validation data to assess suitability of the results for the region, period, and variables of interest for different data applications. Here we provide high-level summaries of some of the key cross-validation metrics, focusing on daily MAE for temperature and precipitation. Many other metrics can be calculated from the raw cross-validation results provided in the dataset, according to user needs.

**Fig. 5:**
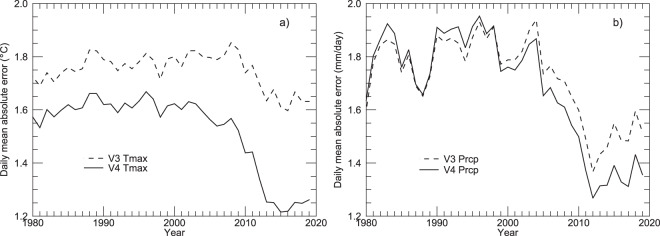
Annual timeseries of daily cross-validation mean absolute error (MAE), averaged over all station days for each year, comparing results for Daymet V4 and Daymet V3. (**a**) MAE for estimation of daily maximum temperature. (**b**) MAE for estimation of daily total precipitation.

The mean daily MAE of Tmin averaged over the 40-year period is about 1.78 °C, and does not show strong trends over time. This is very similar to the same metric for the Daymet V3 database, which is expected since Tmin processing is unchanged from V3 except for differences in the input weather stations. The same daily MAE metric for Tmax is significantly improved compared to V3 (1.52 °C for V4 compared to 1.75 °C for V3), and shows greater improvement in recent years (Fig. 5a). These results are consistent with improvements related to TOO bias correction for Tmax.

Mean daily MAE of Prcp is significantly lower in recent years compared to the early part of the record, consistent with the expansion of the CoCoRaHS network. V4 daily MAE of Prcp is similar to or slightly higher than V3 through about year 2000, after which V4 shows some improvement (lower MAE) compared to V3 (Fig. 5b). This pattern is consistent with the TOO bias correction analysis presented above.

**Fig. 6:**
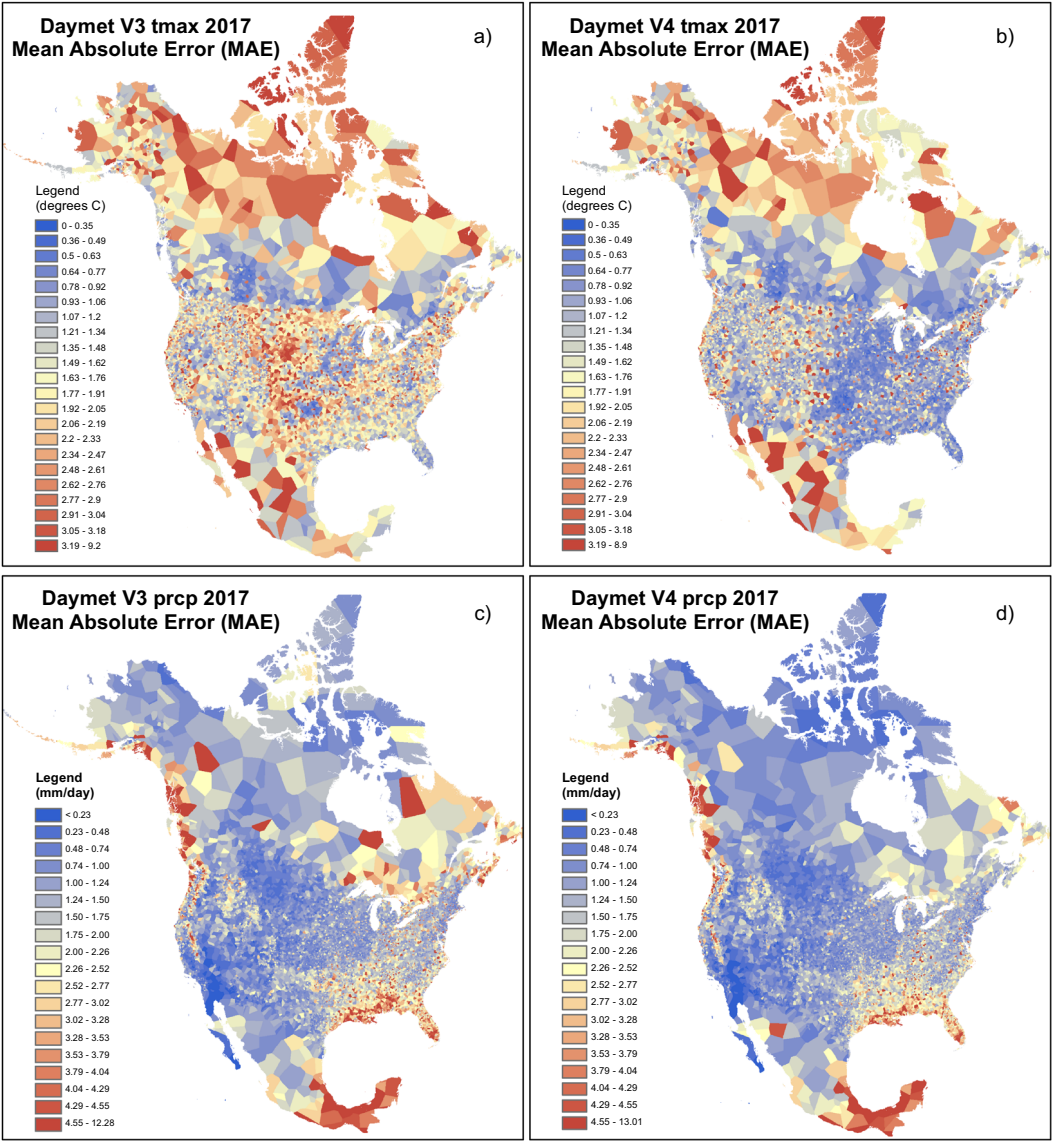
Cross-validation results for all stations, showing daily mean absolute error (MAE) averaged over all days in a single year (2017) and mapped using a nearest-neighbor interpolation to objectively display the influence of varying station density and other factors on data accuracy. Results from the previous version of the Daymet dataset (V3) are shown alongside the latest (V4) results to highlight spatial patterns of improvement based on the new methods described here. (**a**) Annual mean of daily MAE for Tmax, V3. (**b**) Annual mean of daily MAE for Tmax, V4. (**c**) Annual mean of daily MAE for Prcp, V3. (**d**) Annual mean of daily MAE for Prcp, V4.

Variation in station density, terrain, and large-scale atmospheric patterns all contribute to spatial heterogeneity in cross-validation statistics. Introduction of TOO bias correction reduces cross-validation error in regions where different TOO protocols occur in close proximity, such as across the US-Canada border (Fig. 6a,b). Improvements in Prcp cross-validation error are more localized, and are most noticeable in the Great Lakes region and across parts of the eastern seaboard and southeastern US (Fig. 6c,d).

**Fig. 7:**
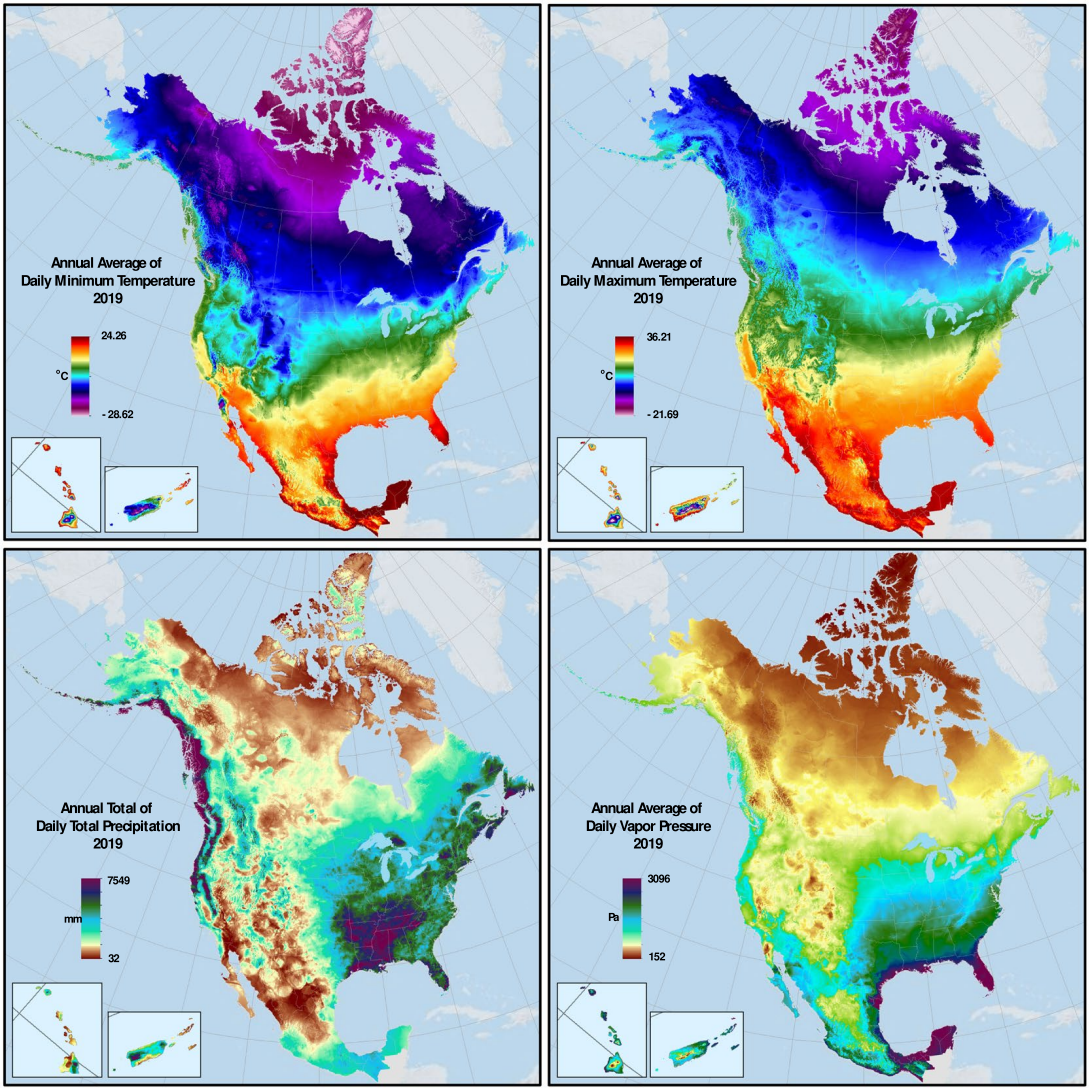
Annual Daymet V4 climatologies for 2019, for Tmax (upper right), Tmin (upper left), Prcp (lower left), and vapor pressure (lower right).

To help evaluate the dataset for the presence of obvious spatial discontinuities or other similar anomalies, we provide a sample of Daymet V4 daily data aggregated to annual climatologies and mapped for 2019, for Tmax, Tmin, Prcp, and the secondary output variable vapor pressure (Fig. 7). These climatologies include the separately generated subregions for Hawaii and Puerto Rico. The climatologies show both large and small-scale features, and visual inspection indicates that there are not obvious spurious patterns or discontinuities.

**Fig. 8:**
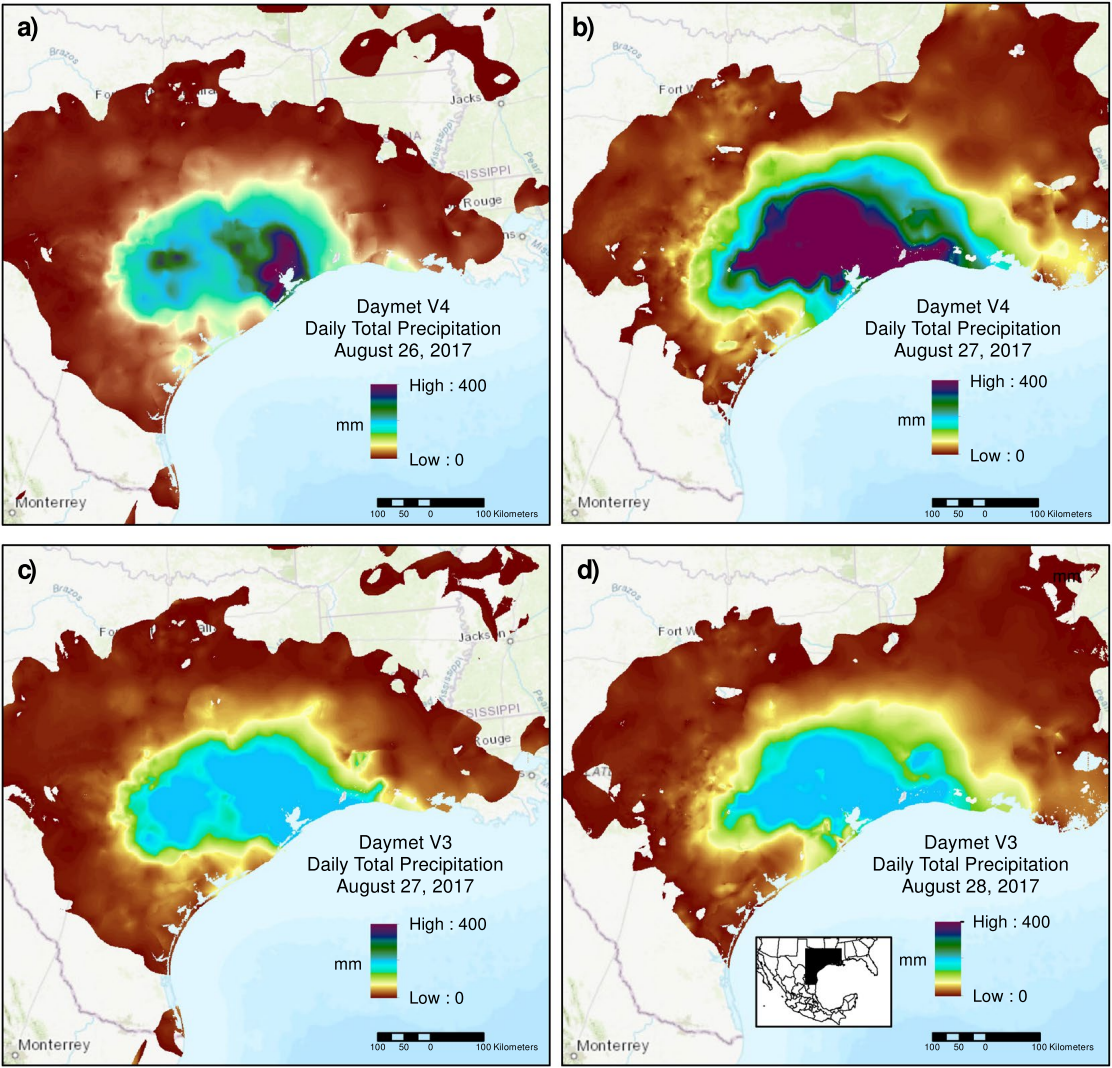
Daily total precipitation for a sub-region that shows landfall of Hurricane Harvey in late August 2017. Panels a) and b) show two days from the Daymet V4 dataset. Panels c) and d) show the corresponding days from the V3 dataset. Inset in panel d) shows the location of detailed region. Note that time-of-observation shifting for precipitation estimates means that August 27 from V3 corresponds best with August 26 from V4, and likewise August 28 in V3 corresponds best with August 27 in V4.

One consequence of changes in the Daymet algorithm for V4 is that hard upper limits on daily precipitation amounts have been removed. This is important in resolving extreme events where more than 200 mm of precipitation might be observed in a day (200 mm/day being the V3 hard limit). We show an example of how removing the upper limit for daily precipitation leads to significantly higher daily precipitation estimates during two days in late August 2017 as Hurricane Harvey was making landfall on the Gulf coast (Fig. 8). The higher daily rainfall totals in V4 are reflective of measurements made, for example, by the local National Weather Service office in Houston, Texas, which showed 370 mm of rainfall on August 26, an all-time record for that station which was broken the following day with 408 mm on August 27.

## Usage Notes

The Daymet project website (https://daymet.ornl.gov) provides links to all of the methods for obtaining Daymet data through the ORNL DAAC. The data are available for direct download through the dataset landing pages pointed to by the dataset DOIs https://daac.ornl.gov/cgi-bin/dataset_lister.pl?p=32. The data are also available through different services, including:

- Thematic Real-time Environmental Distributed Data Services (THREDDS) Data Server (TDS) at https://thredds.daac.ornl.gov/thredds/catalogs/ornldaac/Regional_and_Global_Data/DAYMET_COLLECTIONS/DAYMET_COLLECTIONS.html provides the ability to obtain subsets of Daymet daily surface weather data, monthly climatology, and annual climatology data sets. Instructions for using the THREDDS netCDF subsetting web service can be found at https://daymet.ornl.gov/web_services.html.
- The ORNL DAAC’s Spatial Data Access Tool (SDAT) at https://webmap.ornl.gov/ogc provides visualization and Open Geospatial Consortium (OGC)-compliant web services for the monthly and annual climatologies.
- The Daymet Single Pixel Extraction tool at https://daymet.ornl.gov/single-pixel/ provides a graphical user interface and web services for extracting a time series of Daymet daily surface weather data at a specific location.
- The Daymet Tile Selection Tool at https://daymet.ornl.gov/gridded/ provides a method for getting the daily surface weather data in gridded 2 degree by 2 degree tiles.
- The ORNL DAAC’s Fixed Sites Subsetting Tool at https://modis.ornl.gov/sites/ provides pre-computed time-series visualizations and data downloads (csv and JSON) for approximately 1400 sites in North America which are part of one or more ecological research networks.

All Daymet data are publicly available, without restriction, according to the NASA Earth Observing System Data and Information System (EOSDIS) Data Use Policy at https://earthdata.nasa.gov/earth-observation-data/data-use-policy. A NASA Earthdata Login account is generally needed to download data, available through https://urs.earthdata.nasa.gov. Any person can get an Earthdata Login account through an automated process.

## Data Availability

The source code implementing the core Daymet algorithms (Eq. 2 through 17 and associated text) is available at 10.5281/zenodo.4737573^29^.
